# Artificial Intelligence for Lymph Node Detection and Malignancy Prediction in Endoscopic Ultrasound: A Multicenter Study

**DOI:** 10.3390/cancers17213398

**Published:** 2025-10-22

**Authors:** Belén Agudo Castillo, Miguel Mascarenhas Saraiva, António Miguel Martins Pinto da Costa, João Ferreira, Miguel Martins, Francisco Mendes, Pedro Cardoso, Joana Mota, Maria João Almeida, João Afonso, Tiago Ribeiro, Marcos Eduardo Lera dos Santos, Matheus de Carvalho, María Morís, Ana García García de Paredes, Daniel de la Iglesia García, Carlos Estebam Fernández-Zarza, Ana Pérez González, Khoon-Sheng Kok, Jessica Widmer, Uzma D. Siddiqui, Grace E. Kim, Susana Lopes, Pedro Moutinho Ribeiro, Filipe Vilas-Boas, Eduardo Hourneaux de Moura, Guilherme Macedo, Mariano González-Haba Ruiz

**Affiliations:** 1 Hospital Universitario Puerta de Hierro Majadahonda, 28222 Madrid, Spainana.perez.gs96@gmail.com (A.P.G.);; 2 Centro Hospitalar Universitário São João, 4200-319 Porto, Portugal; 3 Department of Mechanical Engineering, Faculty of Engineering, The University of Porto, 4200-465 Porto, Portugal; 4 Hospital das Clínicas da Faculdade de Medicina da USP, São Paulo 05403-010, Brazileduardoghdemoura@gmail.com (E.H.d.M.); 5 Vila Nova Star Hospital, São Paulo 04544-000, Brazil; 6 Hospital Universitario Marqués de Valdecilla, 39008 Santander, Spain; 7 Hospital Universitario Ramon y Cajal, 28034 Madrid, Spain; anaggparedes@gmail.com; 8 Royal Liverpool Hospital, Liverpool L7 8YE, UK; khoonsheng@gmail.com; 9 NYU Langone Health, New York, NY 10016, USA; 10 Center for Endoscopic Research and Therapeutics (CERT), University of Chicago, Chicago, IL 60637, USA

**Keywords:** artificial intelligence, deep learning, endoscopic ultrasound, lymph nodes

## Abstract

**Background/Objectives**: Endoscopic ultrasound (EUS) is crucial for lymph node (LN) characterization, playing a key role in oncological staging and treatment guidance. EUS criteria for predicting malignancy are imprecise, and histologic diagnosis may have limitations. This multicenter study aimed to evaluate the effectiveness of a novel artificial intelligence (AI)–based system in predicting LN malignancy from EUS images. **Methods**: This multicenter study included EUS images from nine centers. Lesions were labeled (“malignant” or “benign”) and delimited with bounding boxes. Definitive diagnoses were based on cytology/biopsy or surgical specimens and, if negative, a minimum six-month clinical follow-up. A convolutional neural network (CNN) was developed using the YOLO (You Only Look Once) architecture, incorporating both detection and classification modules. **Results**: A total of 59,992 images from 82 EUS procedures were analyzed. The CNN distinguished malignant from benign lymph nodes with a sensitivity of 98.8% (95% CI: 98.5–99.2%), specificity of 99.0% (95% CI: 98.3–99.7%), and precision of 99.0% (95% CI: 98.4–99.7%). The negative and positive predictive values for malignancy were 98.8% and 99.0%, respectively. Overall diagnostic accuracy was 98.3% (95% CI: 97.6–99.1%). **Conclusions**: This is the first study evaluating the performance of deep learning systems for LN assessment using EUS imaging. Our AI-powered imaging model shows excellent detection and classification capabilities, emphasizing its potential to provide a valuable tool to refine LN evaluation with EUS, ultimately supporting more tailored, efficient patient care.

## Simple Summary

Endoscopic ultrasound is fundamental for lymph node assessment, being pivotal in oncological staging and treatment guidance. However, significant limitations are observed when considering EUS criteria for prediction of lymph node malignancy. The authors aimed to develop a YOLO convolutional neural network for artificial intelligence-based prediction of lymph node malignancy during EUS. The model had an overall accuracy over 98% for prediction of lymph node malignancy, with an image processing time that favors its clinical applicability. This is the first study worldwide study evaluating deep learning models for lymph node assessment using EUS imaging, enabling artificial intelligence-assisted EUS has a novel tool for achieving a more accurate and tailored patient management.

## 1. Introduction

### 1.1. Background and Clinical Significance

Distinguishing between malignant and benign lymph nodes (LNs) remains a significant challenge in clinical practice. Conventional imaging techniques, such as magnetic resonance imaging and computed tomography offer limited specificity. Predictive factors, such as the LNs location, distribution, enhancement patterns, and size, have been employed to differentiate between malignant and benign conditions. However, imaging alone often proves insufficient for an accurate diagnosis due to the lack of specificity required for precise LN characterization. Accurate differentiation influences clinical decisions in oncological settings and serves as an essential prognostic factor in determining patient outcomes [1,2]. Conversely, accurate identification of benign LNs supports appropriate treatment decisions and avoids unnecessary procedures and patient anxiety.

EUS provides real-time, high-precision LN evaluation through key morphological features (rounded shape, hypoechoic pattern, sharp margins, size > 10 mm). When combined, diagnostic accuracy reaches 80-100%, yet these features occur in only 25% of malignant LNs, with moderate interobserver agreement (κ = 0.22–0.46) [3,4]. Overall, the prediction of malignancy remains suboptimal, with diagnostic accuracy of only 70–77% [5,6].

EUS elastography (EUS-E) and contrast-enhanced EUS (CE-EUS) are advanced imaging techniques that provide complementary information in LN assessment [7,8,9]. However, these modalities are not universally available and require specific expertise, which can limit their routine application. EUS-guided tissue acquisition—via fine-needle aspiration (FNA) or fine-needle biopsy (FNB)—remains the gold standard for definitive LN characterization [10]. Comparative studies show FNB outperforms FNA in both accuracy (87.62% vs. 75.24%) and sensitivity (84.71% vs. 70.11%) [11]. Nevertheless, limitations persist as some LNs are inaccessible, sampling remains limited, and diagnostic yield depends on endoscopist expertise and cytopathologist interpretation of representative samples [12]. These challenges highlight the need for novel diagnostic approaches.

Artificial intelligence (AI), particularly convolutional neural networks (CNNs), offers a promising solution to overcome the aforementioned limitations. CNNs analyze complex imaging data, identify patterns beyond human perception, and enhance diagnostic consistency. By reducing reliance on subjective criteria, they can mitigate variability in EUS interpretation and improve LN malignancy prediction. AI-assisted models have already demonstrated success in related fields. In pancreatic cancer detection, CNN-based EUS achieved a sensitivity of 95% and specificity of 90%, outperforming conventional diagnostic methods [13]. Similarly, studies in endobronchial ultrasound have shown promising results for LN evaluation, with diagnostic accuracies ranging from 82–84.3% using CNN models combined with expert interpretation [14,15].

Despite advances in other modalities, applying CNNs to EUS for LN malignancy prediction remains unexplored. Bridging this gap could improve diagnostic accuracy and patient outcomes.

### 1.2. Study Objectives

Primary Objective: Assess the effectiveness of a CNN in detecting and classifying LN malignancy using EUS images.

Broader Context: This study leverages a diverse transatlantic dataset from centers in Spain, Portugal, Brazil, the United Kingdom, and the United States, incorporating variations in imaging techniques, devices, and patient populations to enhance model robustness and generalizability.

## 2. Materials and Methods

### 2.1. Study Design, Data Collection and Dataset Preparation

This study analyzed 82 EUS procedures performed across nine centers in five countries (Portugal [*n* = 1], Spain [*n* = 4], the United States [*n* = 2], Brazil [*n* = 1], and the United Kingdom [*n* = 1]) performed between November 2017 and January 2025. All procedures were conducted by experts in EUS, using 4 different EUS processors (Olympus EU-ME2^®^, Olympus EU-ME1^®^, Hitachi Arietta™ 850, Fujifilm Arietta™ 750).

Ethical approval was obtained for all participating centers, ensuring compliance with the Helsinki Declaration. Additionally, anonymized videos from each EUS procedure were processed for labelling, with all personally identifiable information in accordance with General Data Protection Regulations. This study was conducted and reported in accordance with the STARD (Standards for Reporting of Diagnostic Accuracy Studies) guidelines for diagnostic accuracy research.

A CNN was developed to automatically detect and classify LN status as either benign or malignant, using histopathology as the gold standard, based on positive FNA or FNB results, or on surgical specimen analysis. In cases with negative histopathology, a minimum six-month clinical follow-up was required to confirm the benign nature of the lesion. Only iconographic data from biopsied LNs were included in the study. Each frame containing a biopsied LN was annotated by a gastroenterologist and subsequently reviewed by another expert physician. In total, 59,992 frames from processed EUS videos procedures were annotated to develop this AI model, including 27,894 benign and 32,098 malignant frames.

**Figure 1:**
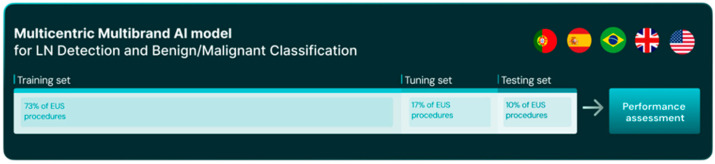
Schematic representation of study design.

The dataset was divided into three mutually exclusive subsets: training (*n* = 60, 73%), tuning (*n* = 14, 17%), and test (*n* = 8, 10%) (Figure 1). To prevent data leakage, each patient—and all associated EUS procedures and frames—was assigned to a single subset only.

A stratified allocation was performed to ensure balanced representation of benign and malignant lymph nodes, participating centers, and EUS device types. The training set included cases from 8 centers and 5 devices (53.2% malignant), while the tuning set included 14 procedures from 7 centers (77.2% malignant). The test set comprised 8 procedures from 3 centers (69.4% benign), all acquired with the Olympus EU-ME2 processor to ensure uniform imaging conditions.

Given the relatively small sample size, this structured split was chosen over a random allocation to enhance model training, maintain subset balance, and ensure independent validation.

### 2.2. AI Model Selection and Development

This work utilized the YOLOv11 (You Only Look Once) framework as the AI model for object detection. The model identifies objects of interest by enclosing them in bounding boxes and assigning corresponding classes, such as “Malignant” or “Benign”. Image pre-processing was performed to omit patient information and ensure uniformization of data. The input image is processed through a deep neural network, which extracts a high-level feature map representing the image. This feature map is then divided into an N × N grid, with each cell responsible for detecting objects whose centers fall within its boundaries. Non-maximum suppression (NMS) is applied to refine the detected bounding boxes. This technique assesses the similarity of nearby bounding boxes using the intersection over union (IoU) metric and confidence score. The confidence threshold and the IoU threshold applied during NMS play a crucial role in optimizing the performance of the YOLOv11 model. These parameters were selected to balance precision and recall in identifying lesions. The analyses were performed with a computer equipped with a double AMD EPYC 7282 16-Core central processing unit (AMD, Santa Clara, CA, USA) and a NVIDIA RTX™ A6000 graphic processing unit (NVIDIA Corporate, Santa Clara, CA, USA).

### 2.3. Performance Evaluation: Lesion Detection and Diagnosis (Classification) Task

The model’s performance was assessed using the test set, focusing on detection and classification tasks independently evaluated on a per-image and per-lesion basis. To comprehensively assess performance, we systematically evaluated all combinations of five thresholds for intersection over union (IoU) and five thresholds for confidence score (0.1, 0.3, 0.5, 0.7, 0.9), resulting in a 5 × 5 grid (25 combinations). For each pair, we computed key metrics including detection rate, sensitivity, specificity, precision, recall, F1-score, accuracy, positive predictive value (PPV), and negative predictive value (NPV). This approach was designed not to select a single optimal threshold, but to explore the model’s behavior across a range of operating conditions and provide a performance spectrum relevant to real-world applications.

**Figure 2:**
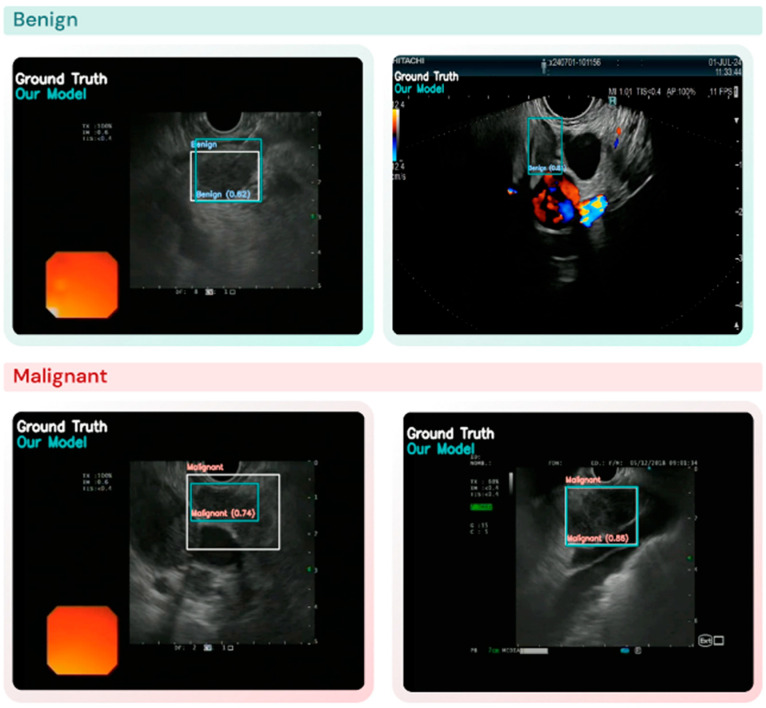
Comparison between YOLO model predictions and ground truth in the detection and differentiation of lymph nodes (LN).

A LN was correctly detected when the model produced a bounding box that accurately enclosed the node in the image (Figure 2). The detection rate was calculated as the number of correctly detected LNs divided by the total number of nodes.

The classification performance was evaluated as sensitivity, specificity, PPV, NPV, and accuracy. We also evaluated the discriminatory capacity of the AI model by calculating the area under the precision-recall curve (AUPRC). Precision-recall curves and mean average precision values were calculated for an IoU threshold of 0.5.

### 2.4. Statistical Analysis

The evaluation of the overall model performance included a comparative analysis of the detection and classification of benign and malignant lesions. The model performance in detecting lesions was reported as detection rates. The model performance in classifying lesions was reported as sensitivities, specificities, PPVs, NPVs, and accuracies with their respective 95% confidence intervals (95%CI).

## 3. Results

### 3.1. Dataset Characteristics

A total of 59,992 annotated frames from 82 EUS procedures were included. Table 1 provides a detailed distribution of frames, exams, devices and number of benign and malignant frames.

The dataset was divided into three sets: the training set included 44,745 frames from 60 exams, with a mean patient age of 63.1 years (SD: 10.5), consisting of 34 males (56.7%) and 26 females (43.3%). The tuning set comprised 7748 frames from 14 exams, with a mean age of 66.1 years (SD: 19.2), equally distributed between males (7; 50.0%) and females (7; 50.0%). The testing set contained 7499 frames from 8 exams, with a mean patient age of 59.0 years (SD: 6.7), also equally distributed between males (4; 50.0%) and females (4; 50.0%). Table 2 details the number of exams per center.

### 3.2. Lymph Node Detection and Classification Performance

**Figure 3:**
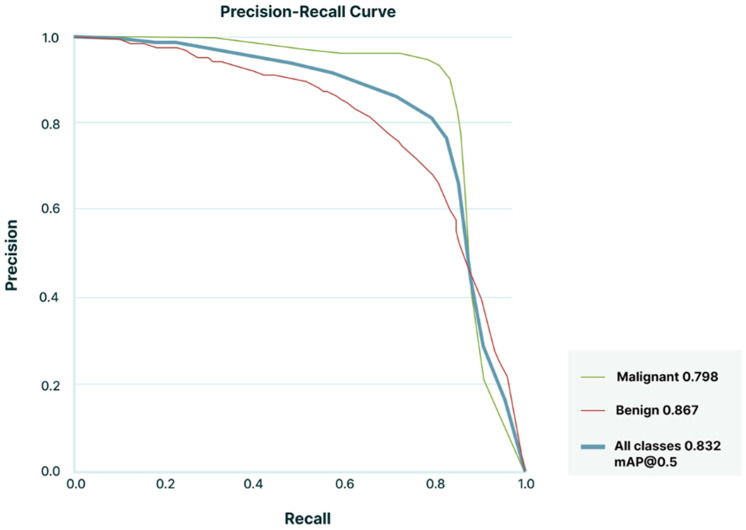
Precision-Recall Curve on LN detection and differentiation. The model achieved a mean average precision (mAP) of 0.82 at an Intersection over Union (IoU) threshold of 0.5 (mAP@0.5) across all classes.

The frame-level detection rates for malignant and benign lesions were 85.4% (95% CI: 80.4–90.3) and 69.1% (95% CI: 60.2–78.0), respectively. Once a LN was successfully detected, the AI model demonstrated comparable sensitivity in correctly classifying malignant and benign lesions, with values of 98.8% (95% CI: 98.5–99.2%) and 96.8% (95% CI: 94.8–98.9%), respectively. The NPV for malignant LNs was 98.8% (95% CI: 98.4–99.2%), while for benign lesions, it was 97.0% (95% CI: 95.0–98.9%). The overall accuracy of the AI model for diagnosing both malignant and benign lesions was 98.3% (95% CI: 97.6–99.1%). Table 3 summarizes the main performance metrics in the automatic detection and classification of LN histology and Figure 3 shows the AUPRC for the AI model during testing set assessment.

## 4. Discussion

To our knowledge, this is the first study to evaluate the performance of a deep learning system for LN assessment using EUS imaging. The developed CNN demonstrated a significant improvement over traditional EUS diagnostic approaches for LN malignancy detection. This proof-of-concept study specifically focuses on the classification accuracy of LNs once visualized by EUS.

Conventional EUS relies on morphological criteria with variable sensitivity and specificity, limited by operator dependency and interobserver variability [5,6]. For instance, while agreement on LN malignancy is considered “good” (κ = 0.65), features like echogenicity (κ = 0.46), borders (κ = 0.43), and shape (κ = 0.35) demonstrate only moderate to fair agreement among experienced endosonographers [6]. Auxiliary techniques like EUS-guided elastography enhance LN evaluation by providing tissue stiffness insights, with reported sensitivity, specificity, and accuracy of 91.8%, 82.5%, and 88.1%, respectively, with interobserver agreement (κ = 0.657) [16]. However, qualitative elastography’s reliance on color patterns (e.g., blue for malignant, green for benign) introduces subjectivity. Quantitative approaches using strain ratios and hue histograms have improved precision, with studies reporting accuracies of around 90% [17,18]. These advancements underscore elastography’s potential, though challenges in standardization and operator dependency remain.

Dedicated CE-EUS improves sensitivity (87.7%) and specificity (91.8%) for LN evaluation, but remains suboptimal, and current guidelines do not recommend routine use [8]. Its utility is further limited when multiple LNs require evaluation, as repeated contrast administrations complicate the procedure.

EUS–guided tissue acquisition remains the reference standard for obtaining definitive LN diagnoses, with reported sensitivities close to 90% and specificities reaching 100% [12,19]. However, its performance is influenced by factors such as location, sampling errors, and histopathological interpretation. False negatives, often due to small lesion size, interposed vascular structures, or severe inflammation, remain a notable limitation. Moreover, in the context of tumor staging, it is common to encounter multiple LNs that may appear suspicious. However, performing a biopsy on each of them is not feasible in most cases.

Our novel AI-driven approach has the potential to address these limitations. By leveraging a YOLO-based CNN model, we demonstrated excellent diagnostic performance, with high sensitivity of 98.8% for malignant and 96.8% for benign LNs, and an overall accuracy of 98.3%. Its ability to detect LNs and to characterize them with high precision (ranging from 96.3% to 99%) adds a layer of objectivity and reliability to traditional EUS workflows. Additionally, the high AUPRC underscores the CNN’s robustness and reliability in predicting malignancy. By reducing reliance on operator expertise, the CNN presents a standardized and scalable approach to LN characterization, particularly in low-resource settings or institutions with less experienced endoscopists [20].

By integrating CNN-based predictions, clinicians can improve the selection of suspicious LNs for tissue acquisition, potentially reducing procedural times and improving diagnostic yield.

A key strength of this study lies in the use of a large and diverse dataset, comprising 59,992 EUS images from 9 international centers. This diversity enhances the model’s robustness, allowing it to generalize better across different patient populations and clinical settings. By leveraging data from varied sources, the CNN demonstrates its adaptability and reliability, which are crucial for widespread clinical adoption.

Another major advantage is the rigorous selection of patients with LNs assessed through the gold standard of tissue diagnosis, incorporating histopathological confirmation via biopsy, surgical specimen analysis, and longitudinal follow-up for benign LNs. This approach ensures high diagnostic accuracy and minimizes the risk of misclassification bias. Recent years have confirmed the superiority of EUS-guided fine-needle biopsy (EUS-FNB) for tissue sampling of solid lesions. However, significant variability in sampling techniques persist [21,22]. Although this was not the aim of the present study, subsequent lines of investigation should compare the results of computer-aided detection and diagnosis to those obtained by EUS-FNB and its different technical variations.

This study fills a critical gap in gastrointestinal oncology diagnostics by introducing a novel, YOLO-based CNN model adapted specifically for EUS imaging. YOLO’s dual capabilities in detection and characterization enable real-time LN analysis, making it uniquely suited for integration into clinical EUS workflows. The ability to identify malignant LNs in real time is particularly advantageous in cases where multiple LNs are present, as the AI model can help triage which lesions warrant further evaluation. The CNN’s high specificity for benign lesions reduces unnecessary biopsies and improves procedural efficiency.

The study’s retrospective design introduces inherent limitations that warrant consideration. Retrospective labeling and bounding box annotations may have introduced inconsistencies due to subjective interpretation of lesion boundaries. Variability in imaging protocols, resolution, contrast settings, and acquisition techniques across the nine participating centers could have affected model performance. In this proof-of-concept study, no data augmentation or image regularization techniques were introduced. Nevertheless, the patient-level data split mitigates the risk of data leakage. Additionally, selection bias may exist since only patients with definitive diagnoses confirmed or extended follow-up were included, potentially underrepresenting ambiguous or atypical histologic cases.

Real-world integration may reveal discrepancies in performance due to variations in operator skill and ultrasound devices. The inclusion of data from a large number of centers provides insight into the potential of the model to operate adequately across variable conditions. However, in this proof-of-concept stage, no per center analysis was conducted. This should be assessed during prospective multicenter validation of the algorithm. Moreover, the test set was relatively small (8 procedures) and employed a single ultrasound processor (Olympus EU-ME2), although it included cases from three independent centers. Larger and more device-diverse test sets are needed to confirm model generalizability.

Although the CNN demonstrated excellent diagnostic accuracy, false negatives and positives remain a challenge, potentially leading to delayed treatment or unnecessary interventions. The use of bounding boxes for annotation, while practical, oversimplifies LN morphology, suggesting that advanced segmentation techniques could improve model precision.

Error analysis identified several contributing factors to misclassification [23,24]:

- Image Artifacts: Artifacts such as noise, shadowing, or low-resolution images are known to contribute to the CNN’s inability to accurately detect and classify LNs. These issues were more prevalent in cases where classified images were taken under suboptimal conditions, such as endoscope movement or in challenging probe positions.
- Class Imbalance: Despite including a large number of patients (and LN images) and efforts to balance the dataset, a slight disproportion in malignant versus benign LNs in certain subgroups may have introduced some performance bias.
- Annotation Challenges: The reliance on bounding boxes for lesion annotation, while practical, may not always capture the full morphological complexity of LNs, leading to incomplete training signals for the model. This limitation could be addressed with more advanced segmentation approaches in future iterations.

Addressing these limitations will require prospective, multicenter studies with standardized imaging protocols, expanded datasets and maintain the inclusion of diverse patient populations. Additionally, implementing techniques such as image preprocessing to reduce artifact effects, improving annotation strategies with precise segmentation tools, and exploring multimodal approaches that integrate imaging with clinical and molecular biomarkers will further enhance model accuracy. Moreover, future studies should integrate ancillary techniques such as elastography and contrast-enhanced EUS, comparing their standalone diagnostic efficiency with that of AI.

Efforts will also focus on developing explainable AI systems to increase clinician trust and facilitate adoption in routine clinical practice [25]. Real-time testing and validation in live EUS settings will further refine the model’s utility and integration capabilities.

This study shows how CNNs have the potential to improve LN detection and characterization using EUS. It fosters further investigation into finding answers on how this technology could be integrated into clinical practice, tested further, and developed to its full potential. If and when integrated in real life EUS workflows, the model has the potential to support clinicians in making quicker and more informed decisions, such as whether a biopsy is necessary and, in that case, which node to sample. To achieve this, future efforts should focus on the creation of software that works smoothly with existing EUS systems (interoperability—FAIR principles), improving response time, so to increase effectiveness during live procedures (hence the choice of YOLO as the object detection model used), and design user-friendly surfaces. While the comparison between the YOLO model and other architecture was beyond the scope of this study, larger studies should provide this comparison. Training programs will also play an important role in helping healthcare providers trust and effectively incorporate AI tools in their daily practice.

Although these results are promising, further research is needed to validate the model in diverse clinical environments. The current dataset, from 9 high-volume centers across 4 countries and 2 continents, supports generalizability; however, testing in other regions and settings is required to confirm consistent performance.

Future models could integrate imaging with clinical, laboratory, or genetic data to improve diagnostic accuracy and clinical utility. Another potential area of interest would be the combination of various CNNs working at the same time to generate a more global and dynamic assessment of the clinical scenario during live procedures. Our group has developed previous CNNs focused on recognition and differentiation of solid and cystic pancreatic lesions [25]; a practical extension of this concept would involve combining these CNNs into a unified system capable of achieving near-complete pancreatic cancer staging (assessing both T and N components) during a single procedure. By integrating different types of data and combining various models, AI tools could move beyond imaging to become part of a broader diagnostic framework, improving patient care and outcomes.

## 5. Conclusions

This study demonstrates the clinical value of a YOLO-based CNN in improving EUS assessment of LNs. Trained on a large, diverse dataset, the model achieved high sensitivity, specificity, and accuracy, reducing operator variability and complementing established EUS and FNA/FNB techniques. Its strong performance supports integration into clinical workflows with potential to improve patient outcomes through more accurate and efficient LN characterization. Further research is needed to validate the model in larger multicenter trials and assess its real-time clinical application. Close collaboration between clinicians and engineers will be essential to fully realize the potential of AI in this field.

## Figures and Tables

**Table 1:** Dataset Summary.

Set	Frames (*n*)	Exams (*n*)	Devices (*n*)	Benign (*n*)	Malignant (*n*)
Training	44,745	60	5	20,922	23,823
Tuning	7748	14	4	1764	5984
Testing	7499	8	1	5208	2291

**Table 2:** Distribution of Exams per Participating Center across Training, Validation, and Test Sets.

Center	Training	Validation	Testing
Center 1	4	1	2
Center 2	38	8	3
Center 3	0	1	0
Center 4	2	1	0
Center 5	1	1	0
Center 6	9	1	3
Center 7	1	1	0
Center 8	3	0	0
Center 9	2	0	0

**Table 3:** Performance metrics of the AI model for benign and malignant lymph node (LN) detection and classification. PPV: positive predictive value; NPV: negative predictive value; F1-score: harmonic mean of sensitivity and PPV.

Metric	Benign (%) [95% CI]	Malignant (%) [95% CI]
Detection rate	69.1 [60.2–78.0]	85.4 [80.4–90.3]
Sensitivity (Recall)	96.8 [94.8–98.9]	98.8 [98.5–99.2]
Specificity	96.3 [95.1–97.6]	99.0 [98.3–99.7]
PPV	96.3 [95.0–97.6]	99.0 [98.4–99.7]
NPV	97.0 [95.0–98.9]	98.8 [98.4–99.2]
Accuracy	98.3 [97.6–99.1]	98.3 [97.6–99.1]
F1-score	96.6 [95.0–98.1]	98.9 [98.4–99.4]

## Data Availability

Data are contained within the article.
